# What Is the Hobbit?

**DOI:** 10.1371/journal.pbio.0040440

**Published:** 2006-12-12

**Authors:** Tabitha M Powledge

## Abstract

The tiny hominid bones, which a joint Australian-Indonesian team unearthed in 2003 on the Indonesian island of Flores, have quickly become as celebrated (and derided) as any find in the tempestuous history of human paleontology.

Who—or what—is Homo floresiensis? The tiny hominid bones, which a joint Australian-Indonesian team unearthed in 2003 on the Indonesian island of Flores, have quickly become as celebrated (and derided) as any find in the tempestuous history of human paleontology. The mystery that shrouds these ancient skeletons, nicknamed hobbits after the diminutive characters in J. R. R. Tolkien's novels, seems to deepen with every study published. Two main camps have emerged, each certain they can settle the question. But many other paleoanthropologists confess they still have no idea.

## H. floresiensis Discovered

The discovery team declared their find a new human species, H. floresiensis, based primarily on a single near-complete skeleton of one very small individual with a very small brain, known as LB1. Compared to H. sapiens, LB1, whose age was estimated from tooth wear at about 30 years, was only one meter tall—about the size of a 4-year-old H. sapiens child—with a brain the size of a newborn's. Although there are also fragments of eight other small individuals, they provide no information about brain size, nor is much skeleton preserved. Nonetheless, they possess a combination of features never before seen in human fossils, which makes it credible that a previously unknown population of people smaller than today's pygmies lived on Flores between 90,000 and 12,000 years ago.

H. floresiensis…could be the first human example of island dwarfing.

Stone tools found at the site raise the possibility that hobbits had culture, even though LB1's brain size would make a chimpanzee sneer. H. floresiensis, the discovery team claimed, could be the first human example of island dwarfing. This phenomenon, thought to be evolution's response to limited resources, is known for other mammals, including dwarf elephants from Flores itself. But this is not the only possible conclusion. A long-awaited paper, which appeared online in Proceedings of the National Academies of Sciences of the United States of America (PNAS) on August 23, 2006, offers a radically different interpretation of these skeletal remains.

## H. floriensis Disputed

The lead author of the PNAS paper is Teuku Jacob, the “Grand Old Man” of Indonesian paleoanthropology. Jacob was not a member of the discovery team, but his lab at Gadjah Mada University in Yogyakarta on Java is home to many hominid fossils discovered in the region. For reasons still unclear, soon after the journal Nature published the discovery papers in October 2004, the hobbit bones were conveyed to Jacob's lab from the Center for Archaeology in Jakarta, about 450 kilometers away.

The result was an international uproar played out in the media. Australian members of the discovery team were irate because their finds were moved without their permission and were no longer available for study. Other paleontologists expressed alarm at what seemed like one group of scientists making off with finds of another. Jacob said he had been invited to take them.

Moreover, Jacob pronounced that the bones did not belong to a new hominid species, but were those of H. sapiens after all. How to account, then, for the diminutive size of LB1? Jacob concluded that LB1 suffered from microcephaly, a disorder resulting in abnormal development of the brain and, often, body. His diagnosis was backed by Indonesian colleagues and also a few Australian and American researchers whom he permitted to study the bones.

After complex negotiations, most of the bones were returned to Jakarta in February 2005. But the uproar revived when discovery team member Michael Morwood of the University of New England (Armidale, Australia) protested that LB1's pelvis was smashed and another hobbit's jaw was broken and repaired clumsily. Jacob responded that the bones were fine when they left his lab.

When the criticisms of Jacob and his collaborators were finally published in August 2006, they reiterated previous claims that the LB1 skull was unnaturally asymmetrical, showing signs of deformity. The paper also reported that many LB1 traits resemble those of Austromelanesians, and some are similar to Rampasasa pygmies living near the dig site at Liang Bua cave. Hobbits are probably related to them, the authors said, and therefore not a new species. Moreover, LB1 was microcephalic. The authors proffered specific anatomic details to establish their case. However, even when researchers agree on details of hobbit anatomy, it seems they can't agree on their meaning.

## Chinless Wonders

Take the chin. Chins mark a skeleton as sapiens; no other hominids have them. Everyone agrees that the two hobbit mandibles lack chins. But the PNAS paper asserts that appearing chinless does not necessarily mean that the hobbits were not H. sapiens. The paper included a photo of a live Rampasasa pygmy with what the authors termed a negative chin.

But paleoanthropologist Jeffrey Schwartz from the University of Pittsburgh (Pittsburgh, Pennsylvania, United States) points out that although having a chin signifies our own species, there are individual exceptions, so chinlessness doesn't say much. “Having a chin provides information. Not having a chin doesn't.”

…it's pretty much the usual fossil furor…

If chinlessness can't illuminate the discussion, what about teeth? Hobbit teeth are a strong reason to think they may not be a new species. Everyone agrees that compared with other hominids, which have big teeth, hobbit teeth are small, like sapiens teeth. Not only are they small, according to paleoanthropologist John Hawks of the University of Wisconsin (Madison, Wisconsin, United States), hobbit teeth are small in the sapiens pattern: first molars are biggest and third molars smallest. H. erectus and Australopiths have different patterns.

Robert Eckhardt of Pennsylvania State University (State College, Pennsylvania, United States), an author of the PNAS paper, argues that chance convergence of traits in different hominid lineages is unlikely to explain these similarities. Coauthor Etty Indriati, colleague of Jacob's at Gadjah Mada, notes that hobbit teeth also share features with Rampasasa pygmy teeth, such as rotation of the premolar, that imply genes in common.

Ralph Holloway, paleoanthropologist at Columbia University (New York, New York, United States), who shares the critics' belief that LB1's brain was pathological, nevertheless was dissatisfied with the PNAS paper. “It's the kind of paper that should be published, but they could have done a better job on it.” Hobbits, he says, should have been compared to several populations. Indriati responds that it is sounder scientifically to compare LB1 with Flores pygmies, its closest neighbors in both space and time.

## The Meaning of Asymmetry

Holloway agrees that LB1's cranium is asymmetrical. But he and others also agree with University of New England co-discoverer Peter Brown that asymmetry could result from being under several meters of deposits for several thousand years, plus the difficulties of recovering and reconstructing bones described originally as like wet blotting paper. Hobbit bones were never fossilized. “To use that asymmetry to make a case for pathology, you know, I don't think that's a very strong argument,” Holloway says.

The PNAS authors reinforced the case for asymmetry with striking composite photographs of LB1's face (see the Figure). They divided right and left sides at the midline and mirrored each side to yield two photos displaying obvious differences in right and left side facial architecture. A “heuristic device to emphasize the left-right differences,” according to Eckhardt. However, says paleoanthropological craniofacial specialist Todd Rae of Durham University in the United Kingdom, “You can know if it's markedly asymmetrical only by comparing it statistically to other specimens that have also undergone the process of burial and recovery—i.e., other fossils.”

**Figure 1:**
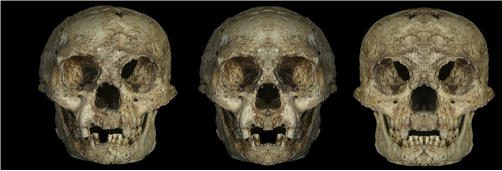
Three Faces of LB1 &brk; The image on the left shows LB1's actual skull. The other two photos are composites made by dividing right and left sides at the midline and mirroring each side to yield two photos displaying obvious differences in right and left side facial architecture. (Photo left: Etty Indriati. Photos center and right: DW Frayer. Courtesy of Proceedings of the National Academy of Sciences of the United States of America, used with permission.)

## The Hobbit's Brain

A March 2005 paper in the journal Science, whose authors include a subset of the discovery team, reported that a virtual endocast of LB1's cranium, which has brain features imprinted on it, suggested that hobbits were not simply miniature versions of sapiens or erectus, but still may have had human-like thinking abilities because the prefrontal cortex and temporal lobes seemed expanded. The region, known as Brodmann area 10, is thought to be the seat of higher cognitive processes like memory, communication, and planning.

By contrast, Holloway, who has also studied an LB1 endocast, says the brain's small size and some other features hint at pathology. Parts of area 10 called the gyri recti seem too thin, he reports, and he has never seen a human endocast so flattened out before, which also suggests abnormality. Like several other researchers, Holloway has tried (and failed) to find direct correspondences between LB1's cranium and those of people with microcephaly. Microcephaly, meaning simply small head, is an umbrella term for a miscellany of conditions with scores of different genetic and environmental causes and myriad manifestations. The most recent study, published online in Anatomical Record on October 9, 2006, concludes that “it is not possible to match any of these syndromes exactly with the LB1 fossil,” although the authors argue that some microcephalic syndromes share features with LB1, including stature, head size, and anomalies of jaw and teeth. As all the studies point out, nothing so far unearthed in the clinical literature or the fossil record matches LB1's peculiar head. Those negatives, of course, don't rule out deformity, especially deformity unique to the hobbits; isolated populations routinely develop distinctive abnormalities.

Debbie Argue of Australian National University (Canberra, Australia) and co-authors of an October 2006 paper in the Journal of Human Evolution say LB1 is probably not microcephalic, and they endorse the designation of a new species. They also say the hobbits are not pygmy-like. They suggest instead (as have others) that, whereas LB1's cranium is not like anything else in the hominid fossil record, some other hobbit bones resemble much older early human (but non-Homo) fossils known only from Africa—the Australopithcines.

## A Very Ancient Mariner?

The original Nature paper had speculated that hobbits might have been a dwarfed descendant of H. erectus. H. erectus was near our size, huge compared with tiny LB1, and members of the discovery team have backed away from that notion. Argue and colleagues concur that H. erectus is an unlikely ancestor because of differences in cranial shape and limb proportions. William Jungers and Susan Larson of the State University at Stony Brook (New York, United States) have analyzed hobbit postcranial material at recent paleoanthropology meetings. They have found limb proportion convergences with Lucy, the most famous member of Australopithecus afarensis. Still, they say, shoulder characteristics more closely match early H. erectus.

At least a genetic link to H. erectus is not geographically outrageous. H. erectus had been in the region for nearly 2 million years; in fact, the type specimen was found more than a century ago on the Indonesian island of Java.

What is a hobbit?

But, unlike big-brained erectus, whose brain was 75% of the size of ours, there is no evidence whatever that small-brained Australopiths (35% the size of ours) ever ventured out of Africa—let alone invented boats and sailed off to Indonesia more than 3 million years ago. The Argue et al. paper speculates on ways very early hominids might have migrated to the region. Something like this astounding seagoing scenario—a really, really ancient mariner—seems to be required if H. floresiensis had Australopith ancestry.

Hawks speculates that this quandary might go away if we knew more about the environmental and developmental factors that determine human body size. “We have no idea what explains body size, and the hypotheses about this are not compelling.”

Maybe LB1's pelvis looks Australopithecine, Hawks suggests, not because the hobbits descend from a seafaring Lucy, but because that's what the pelvis of a very small human biped looks like. “It has the virtue of not depending on external events to explain it,” Hawks says. “It's just a consequence of being small. You still have to explain why they're small.” If we knew why growing big or small was advantageous, we might learn whether island dwarfing applies to people as well as elephants.

Richard Potts of the Smithsonian Institution (Washington, DC, United States) suggests something similar. What would happen, he wonders, if an early, smaller-brained erectus was subjected to island dwarfing? Would ancestral Australopithecine features emerge, perhaps as a result of changes in fetal development? “We'd like to know that, but you know, we just don't.”

## Were Hobbits Toolmakers?

The debate over the hobbit brain has been fueled by stone tools found at the site. A commentary accompanying the original two papers suggested that some were like tools associated elsewhere with modern humans. This suggested to others that hobbits had a complex culture.

Stone tool experts, however, say the tools are not particularly sophisticated. They are simple, made using techniques that hominids have used for millions of years and humans are still using today.

A team led by Adam Brumm of Australian National University has tried to show that hobbits were part of a line of Flores tool makers who had been chipping sharp flakes from stone for upwards of 700,000 years. The researchers based their analysis on more than 500 stone artifacts excavated at another Flores site 50 kilometers from Liang Bua and dated between 800,000 and 740,000 years ago. No erectus remains have been found on Flores so far, but paleoanthropologists assume they made and used the tools. The paper compared the very old tools with those found at Liang Bua and noted specific similarities.

The Flores tools are unique but also simple, according to Potts. “The tools we see on Flores, the older as well as the younger, cannot be exactly matched with anything that's known anywhere else” he says, “but the overall strategy of the technology and the overall size of the materials is consistent with what we find in mainland China.”

Harold Dibble of the University of Pennsylvania Museum agrees. They would fit perfectly at a million-year-old site, he says, but also are a type made by native Australians today. “These are simple tools,” says Dibble, “Could they have been made by a small brain? Sure.”

## The Politics of Hobbitry

H. floresiensis debates have been marked by a degree of acrimony that may seem excessive and even a bit scandalous to outsiders. But a number of paleoanthropologists opine that it's pretty much the usual fossil furor—even though it's been punctuated by public name-calling, a high level of rancor, and exceptionally gaudy episodes like the hobbit bones' unanticipated travels.

“I don't think there's anything special about this dispute except that it's taking place in a particular cultural context of Indonesian politics, which science gets drawn into, just as it does in every other country, at least certain fields of science,” says Potts. “There are aspects of etiquette and the way of treating other people that may represent a bit of a clash between the Australian principal investigators and the Indonesian science community, which tends to be a little bit less, well, freelance about such things. I think that there may be a bit of a culture clash there.”

There are also other theories about LB1 and compatriots. Holloway, for example, says he hasn't given up on his notion that hobbits may have been taken care of and kept as pets by other Flores H. sapiens, “sent out to fetch the wood or the occasional dead rat, bring it back, whatever!”

The most extreme proposition comes from Schwartz: “It's an interesting assemblage of bits and pieces that probably represent different kinds of hominids and maybe even some non-hominids,” he argues. “It's more interesting to me that there might be these different morphologies represented, and the implications of that, than to do a Rube Goldberg hominid and say, ‘Look how weird this is.’”

“To be very blunt, this is just stupid,” says co-discoverer Peter Brown. For example, he explains, LB1's arm articulates with the skeleton, meaning they are from the same individual and not different taxa. But Brown thinks LB1's skeletal proportions and brain size are unlikely to be due to dwarfing of H. erectus. Instead, the most likely ancestor of H. floresiensis was small-bodied and small-brained. “This is not the same as saying the ancestor was an Australopithecine.”

DNA analysis might help, but prospects are gloomy. The site is hot and wet, perfect for destroying genetic material. There's been at least one effort to find DNA in hobbit bone, carried out in the lab of ancient DNA specialist Svante Pääbo of the Max Planck Institute (Leipzig, Germany). It failed.

So on present evidence, the debate about whether hobbits were a different kind of human or simply deformed can't be settled. It is even possible that both sides are partly right. Hobbits are so different from anything else in the hominid record that calling them a new human species is by no means crazy. But even some scientists who buy the new species argument think there may have been something wrong with LB1 and that the much-disputed brain is indeed abnormal.

What is the hobbit? “I don't know,” Holloway admits, and he has plenty of company. But, he observes, the puzzling hobbits don't change the major outlines of human evolution. “I think it's sort of a side issue.”

## References

[pbio-0040440-b001] Argue D, Donlon D, Groves C, Wright R (2006). Homo floresiensis: Microcephalic, pygmoid, Australopithecus, or Homo?. J Hum Evol.

[pbio-0040440-b002] Brown P, Sutikna T, Morwood MJ, Soejono RP, Jatmiko (2006). A new small-bodied hominin from the Late Pleistocene of Flores, Indonesia. Nature.

[pbio-0040440-b003] Brumm A, Aziz F, van den Bergh GD, Morwood MJ, Moore MW (2006). Early stone technology on Flores and its implications for Homo floresiensis. Nature.

[pbio-0040440-b004] Falk D, Hildebolt C, Smith K, Morwood MJ, Sutikna T (2005). The brain of LB1, Homo floresiensis. Science.

[pbio-0040440-b005] Jacob T, Indriati E, Soejono RP, Hsu K, Frayer DW (2006). Pygmoid Australomelanesian Homo sapiens skeletal remains from Liang Bua, Flores: Population affinities and pathological abnormalities. Proc Natl Acad Sci U S A.

[pbio-0040440-b006] Mirazón Lahr M, Foley R (2004). Human evolution writ small. Nature.

[pbio-0040440-b007] Martin RD, Maclarnon AM, Phillips JL, Dobyns WB (2006). Flores hominid: New species or microcephalic dwarf?. Anat Rec A Discov Mol Cell Evol Biol.

